# Promoting physical therapists’ use of research evidence to inform clinical practice: part 2 - a mixed methods evaluation of the PEAK program

**DOI:** 10.1186/1472-6920-14-126

**Published:** 2014-06-25

**Authors:** Julie K Tilson, Sharon Mickan, Jonathan C Sum, Maria Zibell, Jacquelyn M Dylla, Robbin Howard

**Affiliations:** 1 Division of Biokinesiology and Physical Therapy, University of Southern California, 1540 Alcazar St., CHP 155, Los Angeles, CA 90089, USA; 2 Nuffield Department of Primary Care Health Sciences, University of Oxford, Radcliffe Observatory Quarter, Oxford OX2 6GG, UK

**Keywords:** Knowledge translation, Evidence based practice, Education, Post-graduate training, Physical therapy, Mixed methods

## Abstract

**Background:**

Clinicians need innovative educational programs to enhance their capacity for using research evidence to inform clinical decision-making. This paper and its companion paper introduce the Physical therapist-driven Education for Actionable Knowledge translation (PEAK) program, an educational program designed to promote physical therapists’ integration of research evidence into clinical decision-making. This, second of two, papers reports a mixed methods feasibility study of the PEAK program among physical therapists at three university-based clinical facilities.

**Methods:**

A convenience sample of 18 physical therapists participated in the six-month educational program. Mixed methods were used to triangulate results from pre-post quantitative data analyzed concurrently with qualitative data from semi-structured interviews and focus groups. Feasibility of the program was assessed by evaluating change in participants’ attitudes, self-efficacy, knowledge, skills, and self-reported behaviors in addition to their perceptions and reaction to the program.

**Results:**

All 18 therapists completed the program. The group experienced statistically significant improvements in evidence based practice self-efficacy and self-reported behavior (p < 0.001). Four themes were supported by integrated quantitative and qualitative results: 1. The collaborative nature of the PEAK program was engaging and motivating; 2. PEAK participants experienced improved self-efficacy, creating a positive cycle where success reinforces engagement with research evidence; 3. Participants’ need to understand how to interpret statistics was not fully met; 4. Participants believed that the utilization of research evidence in their clinical practice would lead to better patient outcomes.

**Conclusions:**

The PEAK program is a feasible educational program for promoting physical therapists’ use of research evidence in practice. A key ingredient seems to be guided small group work leading to a final product that guides local practice. Further investigation is recommended to assess long-term behavior change and to compare outcomes to alternative educational models.

## Background

The World Confederation for Physical Therapy asserts that physical therapists have a responsibility to integrate research evidence into practice as a foundation of patient care [1]. While most physical therapists embrace this concept in principle, the reality of integrating research evidence into everyday clinical practice has proven challenging [2-6]. One potential method for addressing this problem is the use of theoretically founded, evidence-based educational programs to improve therapists’ capacity and proclivity to use research in practice. This, the second of two companion papers, introduces the Physical therapist-driven Education for Actionable Knowledge translation (PEAK) program – an educational program designed to promote physical therapists’ integration of research evidence into clinical decision-making at the individual and organizational level.

In the companion to this paper [7] we describe the pedagogical foundations, frameworks, and research evidence used to develop the PEAK program. The program’s pedagogy is based on social cognitive theory [8] and adult learning theory [9]. Further, the organizational implementation of research evidence is informed by the Promoting Action on Research Implementation in Health Services (PARiHS) [10] and Knowledge to Action cycle [11] frameworks for knowledge translation (KT). Finally, previous work identifying successful educational models for promoting the use of research in practice were consulted [12-14] as well as useful descriptions of barriers to research implementation [2,5,15].

**Figure 1:**
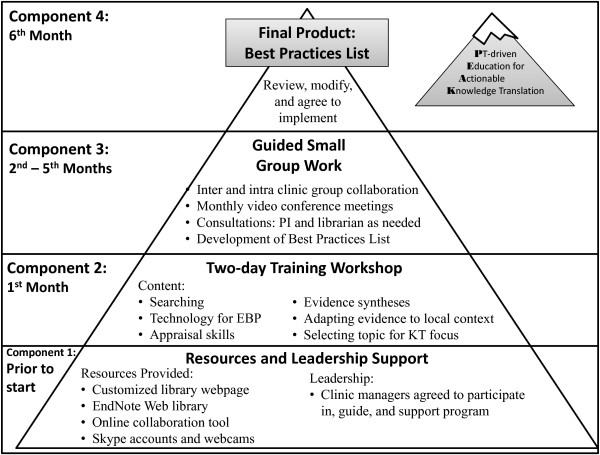
**Timing and integration of components of the Physical Therapist-driven Education for Actionable Knowledge Translation (PEAK) program (figure reads from bottom to top).** The program started with garnering support from clinic managers and placing links to technology resources at each facility’s computer work stations. Next, participants attended a two-day workshop addressing evidence based practice (EBP) and knowledge translation (KT) skills. Five months of guided small group work followed as participants developed the Best Practices List. In the final month, the Best Practices List was reviewed by unaffiliated expert faculty. Finally, after multiple rounds of revisions, all participants agreed to implement the Best Practices List in their clinical practice. (Tilson J, Mickan S, Sum J, Zibell M, Dylla J, Howard R: Promoting physical therapists’ of research evidence to inform clinical practice: part 1 – theoretical foundation, evidence, and description of the PEAK program. BMC Med Educ 2014 14:125.

The 6-month PEAK program consists of four consecutive and interdependent components described in detail in the companion paper and illustrated in Figure 1[7]. The PEAK program is multifaceted and participant-driven making it an inherently complex intervention [16]. Recent advice about designing and evaluating complex interventions suggest that it is important to evaluate the design and its implementation before testing it more rigorously in a randomized controlled trial [17]. This manuscript reports a feasibility study of the PEAK program among a cohort of physical therapists practicing at the University of Southern California (USC). The purpose of this study was to assess the feasibility of the PEAK program with respect to practical implementation, participant reaction, and potential for association with change in participants’ evidence based practice (EBP) attitudes, self-efficacy, knowledge and skills, and self-reported behavior.

## Methods

### Design

A mixed methods “triangulation design model” [18] was adopted to simultaneously collect and analyze quantitative and qualitative data from a single cohort of physical therapists. As quantitative and qualitative data were compared, a deeper level of understanding of the key components of the program and their impact on physical therapists’ clinical practice was evident. This is important to establish the feasibility of the program and to facilitate a deeper evaluation of the underpinning theoretical foundations.

### Procedure

#### Recruitment

Therapists practicing in one of three geographically dispersed USC patient care centers (2 outpatient; 1 inpatient) were invited to participate through staff meetings and individual email. Therapists were required to have a minimum of 6 months clinical experience, be providing patient care at USC at least 20 hours per week, be able to attend both days of an introductory workshop, and be willing to commit to study activities at least 1 hour per month for 6 months. Therapists that also served as onsite clinic managers were included as long as they met the inclusion criteria. The study was approved by the USC Health Science Campus Institutional Review Board (HS-10-00593) and all participants gave consent to participate.

#### PEAK program

The PEAK program, described in detail in this paper’s companion manuscript [7], was 6 months in duration and consisted of four consecutive, interdependent components: 1) securing resources and leadership support; 2) a two-day training workshop; 3) guided small group work to develop a locally relevant list of evidence-based actionable behaviors – the “Best Practices List”; and 4) review, revision, and agreement to implement the Best Practices List (Figure 1).

All components of the PEAK program supported a participant-driven learning experience: to work as a group to generate a Best Practice List around a common, participant-selected clinical area. The Best Practices List is a locally generated list of evidence-based, actionable behaviors that participants agreed (as a group) to implement in their clinical practice. Participants self-organized into small groups to review literature and generate evidence-based actionable behaviors. The actionable behaviors were reviewed and revised through a process of peer and expert review until all participants felt that they could implement the Best Practices List in practice [7].

#### Evaluation of the PEAK program

**Figure 2:**
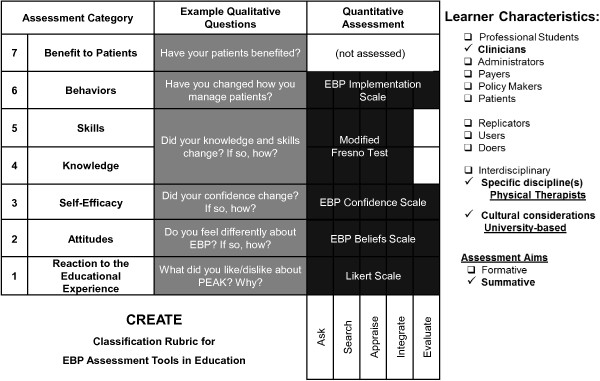
**The Classification Rubric for EBP Assessment Tools in Education (CREATE) framework provided the structure for outcome assessment.** Left column contains seven CREATE assessment categories; middle column contains simplified examples of the types of questions asked in interviews and focus groups to assess participants’ opinions in each category; right column contains standardized assessment tools used to quantitatively assess change in six of seven categories (shading indicates the EBP steps assessed by each tool). (Modified with permission from Tilson J, Kaplan S, Harris J, Hutchinson A, Ilic D, Niederman R, Potomkova J, Zwolsman S: Sicily statement on classification and development of evidence-based practice learning assessment tools. *BMC Med Educ* 2011, 11:78).

The Classification Rubric for EBP Assessment Tools in Education (CREATE) model [19] (Figure 2) provided the theoretical framework for evaluating the feasibility of the PEAK program. CREATE was designed to guide comprehensive and systematic evaluation of complex educational programs like PEAK. It identifies seven important categories for evaluating EBP and KT educational curricula. We used CREATE as the foundational model for selecting quantitative outcome measures and qualitative interview questions to evaluate the PEAK program.

#### Quantitative assessment

Participants completed four standardized assessments collated into one computer-based survey immediately before and after participating in the educational program. This evaluation will focus on changes observed immediately following the PEAK program.

Attitudes toward EBP were assessed using the attitudes items from the EBP Beliefs Scale. The 16-item EBP Beliefs Scale measures EBP attitudes and self-efficacy and has demonstrated construct and criterion validity among practicing nurses [20]. To exclusively measure attitudes about EBP, we summed responses to six Likert-type items from the EBP Beliefs Scale (1,4,5,9,11,13); higher scores (total possible = 30) indicate more positive attitudes. Because it has established face and content validity among healthcare professionals, including physical therapists, the Evidence-based Practice Confidence (EPIC) Scale was used to assess self-efficacy for EBP [21]. EPIC consists of 11 items with responses ranging from 0 to 100% confidence (in 10 percentile increments); responses are averaged to generate a mean confidence between 0 and 100%. EBP knowledge and skills were assessed with the 13-item modified Fresno Test (mFT) which has demonstrated reliability and content and construct validity among physical therapists [22]. The mFT consists of open-ended questions graded with a standardized scoring rubric and results in scores from 0 to 224 with higher scores representing better knowledge and skills. Self-reported EBP behavior was assessed using the EBP Implementation Scale which has demonstrated construct and criterion validity among nurses [20]. The 18-item EBP Implementation Scale assesses implementation of EBP and the collection, analysis, presentation, and reaction to patient data. Because the PEAK program did not ask participants to collect, analyze, present, or react to patient data, 5 items addressing these behaviors (5, 7, 15-17) were not relevant to our study and risked masking any observable changes in self-reported EBP behavior. Therefore, to exclusively measure self-reported behaviors associated with the 5-steps of EBP, we summed responses to 13 items from the EBP Implementation Scale (1-4,6,8-14,18); higher scores (total possible = 65) indicate greater frequency of EBP implementation.

Participants were asked to rate their participation in developing the Best Practices List and to rate the educational value of 11 elements of the PEAK program [2-day workshop, USC medical library resources, customized library webpage, Backpack™ online collaboration tool, local Skype™ access (with webcams), EndNote Web® library, monthly video conference meetings, small group tutorial sessions, access to study librarian, intra-clinic collaboration, inter-clinic collaboration, developing the Best Practices List] on a 5-point likert-type scale. The complete assessment consisted of 70 individual items and was expected to take 60-75 minutes. Scoring was computerized with the exception of the mFT. A trained, blinded assessor scored the mFT.

#### Qualitative assessment

Participants attended either a face-to-face semi-structured interview (1 participant:1 interviewer) or focus group (3-4 participants:1 interviewer) within 2 weeks of completing the PEAK program. A common interview template was developed to explore the range of experiences and subjective reactions to the PEAK program (Appendix A). Questions addressed all seven categories of the CREATE framework. Participants were initially asked to describe their own engagement in, and reaction to, the PEAK program. They were then facilitated to describe the impact of the program on their EBP attitudes, self-efficacy, knowledge, skills and practice behaviors. They were also asked to consider, from their professional experience, whether the program provided a benefit to patients. Finally, they were asked to comment on the feasibility of transferring the PEAK program to other clinical settings (e.g. other institutions and/or patient populations).

Individual interviews and focus groups were conducted by an independent and experienced investigator. The investigator explained to participants that the purpose of the interview was to understand the feasibility of the program and that both supportive and critical comments were welcome. Clinic manager participants were not interviewed with non-manager participants. Interviews and focus groups were conducted at USC. Individual interviews averaged 39 minutes (SD = 14; range = 20-76 minutes) and focus groups averaged 66 minutes (SD = 13; range = 58-78 minutes). All interview sessions were audio taped and transcribed in full. Participants were assigned ID numbers and identifying comments were removed from transcripts to ensure participant anonymity.

### Analysis

Using a triangulation design model, quantitative and qualitative data were first analyzed independently (in parallel). This parallel analysis was followed by integrated discussion and analysis between two authors (JT, SM) to achieve concurrent triangulation [23]. All analyses used the CREATE model as the organizing theoretical framework.

#### Independent quantitative analysis

Change in standardized quantitative assessments was assessed using paired two-tailed t-tests for normally distributed data (Shapiro-Wilk of p > 0.05). When the normality assumption was met, parametric tests were used for likert-type scales (EPIC, EBP Beliefs and Implementation Scales) based on the fact that each scale represents an underlying continuous concept and has relatively equal intervals [24]. Alpha was set at 0.05 and 95% confidence intervals were calculated. Because this was a feasibility study, only complete data sets were analyzed. Quantitative analyses were conducted using SPSS 18.0.

#### Independent qualitative analysis

All participants were sent their own electronic transcripts for clarification and validation and no changes were requested. Text from all interviews was read and allocated to the appropriate categories on the CREATE framework. The initial coding process was piloted independently by two authors (JT, SM) across four transcripts. Differences in coding were discussed and a final coding system agreed upon that included additional topics beyond the CREATE framework. All transcripts were independently coded by two authors using NVivo software (QSR International). Coding differences were resolved through discussion. It was noted that for the last few interviews, no new ideas emerged, suggesting that saturation was reached after 11 individual interviews and 3 focus groups.

#### Integrated data analysis

Following independent analyses, an iterative process of comparison and further analysis was conducted to integrate the quantitative and qualitative data sets. Preliminary qualitative themes suggested that there might be important differences in participant responses on the standardized questionnaires depending on which of the 5 EBP steps a particular item addressed. We anticipated that item-by-item analysis of standardized assessments that showed overall statistical change would provide exploratory information about participants’ responses across the 5 steps of EBP. Hence, post-hoc Wilcoxon signed-rank tests were conducted for individual items when a statistically significant change in the entire assessment was observed (EPIC and EBP Implementation scales). With this new quantitative information, concurrent thematic analysis of qualitative data continued consistent with recommendations by Pope and colleagues [25]. Explanatory themes were noted as repetitive clusters of meaning that, when combined with quantitative results, offered insight into the PEAK program’s feasibility.

#### Researcher reflexivity

Both investigators responsible for analyzing the qualitative data have academic appointments with responsibility for teaching and facilitating the implementation of EBP and KT. One investigator (JKT) was the primary developer of the PEAK program. Both investigators used their experience educating healthcare practitioners in EBP to inform the analysis. They remained open to identifying positive and negative feedback and to identifying expected and unexpected explanations. Authors were sensitive to the complexity of this educational program and recognized that negative feedback provided opportunity for improvement.

## Results

### Participants

Eighteen physical therapists met inclusion criteria and agreed to participate (Table 1). Participants selected the clinical topic of ‘lumbar spine conditions’ and organized themselves into small groups around five sub-topics [Sub-topic (number of participants): outcome measures (6), stenosis (5), spine tumors (5), non-specific low back pain (3), and post-surgical (3); four participants chose to participate in two small groups]. All participants completed all baseline and immediate post-program quantitative assessments (no missing data) and all participated in an interview or focus group. Pre-post quantitative results were normally distributed for all standardized assessments.

**Table 1:** Participant characteristics

Variable*	
N	18
Age, mean (range)	34.7 (27-51)
Years in practice, mean (range)	7.7 (2-20)
APTA member	15 (83.3%)
**Professional designation**	
Staff PT	16 (77.8%)
Clinic manager	4 (22.2%)
**Highest degree**	
DPT	15 (83.3%)
Masters	3 (16.7%)
**Clinical hours per week**	
11-20 hours†	3 (16.7%)
21-30 hours	3 (16.7%)
31-40 hours	8 (44.4%)
>40 hours	4 (22.2%)
**Primary clinic setting**	
Outpatient	10 (55.5%)
Inpatient acute	8 (45.5%)

*Variables are reported as count and percentage unless otherwise noted.

†Three therapists initially met the inclusion criteria of >20 clinical hours per week but experienced a decrease in clinical hours during the course of the study due to changes in responsibilities.

*Abbreviations*: PT, Physical Therapist; DPT, Doctor of Physical Therapy; APTA, American Physical Therapy Association (primary professional association for US physical therapists).

### PEAK feasibility

#### Reaction to the educational experience

Ten of eleven elements of the PEAK program were rated as having ‘good’ or ‘excellent’ value by at least 80% of participants. Of note, ‘Local Skype™ access with webcams’ was rated as having ‘good’ or ‘excellent’ value by just 47% of participants.

Participants had consistently positive descriptions of their reaction to the PEAK program. They particularly enjoyed the collaborative nature of the small group work.

“I think it was good to collaborate with each other and [for] the final product we were going through each of the behaviors and discussing that as a group. I think that was the most beneficial.” (Participant [P] 18)

They described the opportunity to collaborate with people from different settings and with diverse expertise as empowering and motivating. They emphasized the benefits of working with their group toward the common goal of providing consistent and high quality patient care.

“I think we all felt accountability for each other that we had this group project that we had to work together for that was going to improve the way we deliver care. And I feel like pretty much everyone was dedicated to that.” (P 10)

Participants were invested in the ultimate goal of developing the Best Practices List and this was seen to be an important unifying feature of the program.

“Developing a best practices guideline, going through the literature, looking at clinical guidelines, and actually every single person agreeing that these are good, these need to be used.” (P1)

The use of an online collaboration tool (i.e. Backpack™, 37 Signals, LLC) to set and achieve group goals was seen as important.

“Backpack was almost like Facebook, where it kind of provides us this medium to see what everybody else is thinking and sharing information.” (P14)

A small number of participants, without experience using similar online tools, reported that they either struggled to learn them or simply chose not to try.

“I didn't participate as much in this group as much as I think I could have otherwise… I don't have the time to keep up with that technology.” (P8)

Additionally, there was common frustration (for all) about frequent technical problems during monthly Skype™ video conferences.

“And those Skype meetings made it really easy although we had a lot of Skype™ problems. … We spent an hour trying to get everybody online. So that was a little frustrating.” (P17)

Despite these technical challenges, monthly large group meetings provided a venue for regular progress reports and accountability.

“It’s great to see how all the different clinicians across the board can get together and actually focus on clinical practice. And it’s great to see how therapists in different settings can contribute.” (P1)

Most participants did PEAK work from home or before or after work and many reported struggling to find time to meet with group members. They found it frustrating and burdensome, but felt that the time spent was an important investment in the quality of care provided to patients.

“It takes more time and its more energy and all that. But really, you know, you're doing the best thing for your patients. ” (P4)

Finally, when questioned about whether any components of the program were superfluous, participants were unanimous that all components were necessary. In addition, participants valued the high levels of managerial support. While it was difficult for managers to allocate specific time for this program, they were seen as understanding of the needs of the program and willing to give participating clinicians a degree of flexibility in their work practices.

“The managers here definitely are supportive of implementing this [but] from a time perspective, are challenged.” (P5)

#### Attitudes

The PEAK program was not associated with a statistically significant change in knowledge and skills as measured by the mFT (p = 0.10; Table 2). After the program, the cohort had comparatively high scores for knowledge and skills associated with searching (62-84% of possible points) and low scores for items requiring statistical knowledge and skill (5-29% of possible points).

**Table 2:** Quantitative results

**Domain**	**Assessment**	**Baseline**	**6-months**	**Change**	**p-value**
		**Mean (SD) % of scale**		**Mean (95% CI) % improvement**	
Attitudes	EBP Beliefs Scale [6 attitude-specific items, high score = 30]	24.5	25.1	0.61	0.22
		(2.0)	(2.1)	(-0.39-1.62)	
		81.6%	83.7%	2.4%	
Self-Efficacy	Evidence-based Practice Confidence Scale [11 items, high score = 100%]	54.9	72.8	17.9	<0.001
		(13.1)	(13.7)	(13.5-22.4)	
		54.9%	72.8%	32.6%	
Knowledge and Skills	Modified Fresno Test [13 items, high score = 224]	114.72	124.2	9.5	0.10
		(35.1)	(34.8)	(-1.8-20.8)	
		51.2%	55.4%	8.2%	
Behavior	EBP Implementation Scale [13 study-specific items, high score = 65]	25.0	29.8	5.1	<0.001
		(8.1)	(10.2)	(1.3-2.2)	
		38.5%	45.8%	20.4%	

*Abbreviations*: SD, Standard Deviation; CI, Confidence Interval; EBP, Evidence Based Practice.

In fact, the PEAK program was seen as a way for participants to demonstrate and maintain their positive attitudes toward using research evidence in their clinical practice. Most described having a strong internal professional drive to stay up-to-date and appreciated being challenged to build their skills and become more efficient. For many, this was seen as both a personal and a professional commitment that facilitated quality patient care.

“I definitely think that evidence based practice is a very essential component of patient management. I think it’s the gold standard of patient care and so something I take seriously and I understand the importance of it” (P14)

#### Self-Efficacy

**Figure 3:**
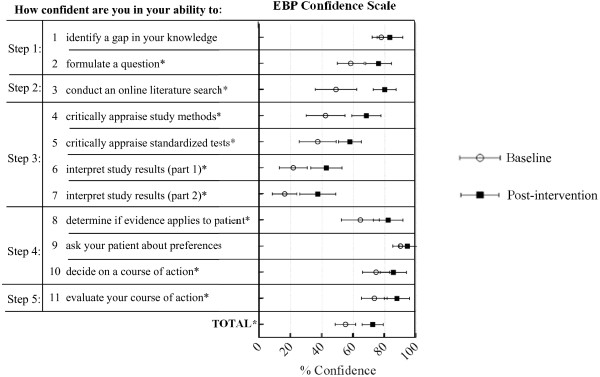
**Illustrates pre-post group mean scores on individual items of the Evidence Based Practice Confidence (EPIC) Scale.** Evidence based practice step (1-5) corresponding to each item is indicated on the left. Open circles indicate baseline mean, closed boxes indicate post-program mean, horizontal bars indicate 95% confidence intervals. ‘*’ indicates statistically significant differences in *post-hoc* analysis.

The PEAK program was associated with a significant increase in self-efficacy for EBP (p < 0.001; Table 2). Mean EPIC scale scores improved from 65.3% to 82.9%. Post-hoc analysis showed statistically significant improvements on all but two individual EPIC items (Figure 3). Notably, despite statistically significant gains, self-efficacy for interpreting study results (Figure 3: items 6 and 7) was still low (<50%) at follow-up.

Participants described improved confidence for accurately and efficiently searching for the best available research evidence. They expressed new found confidence using a variety of tools and had increased expectations that their searches would yield useful information.

“And I feel like I can better access the literature very efficiently with those patients. I'll do it on my phone or very, very quickly. And so I feel like I do that more because I'm more efficient. Whereas before I would go, oh, well, that might take me a little while.” (P5)

Participants also reported new confidence making decisions about how to integrate research evidence into patient care. This seemed to come from a combination of personal success in identifying relevant research and from opportunities to discuss the integration of research evidence with their peers. Participants also described feeling more confident sharing study results with patients.

“I think confidence is a huge validation that you're doing the right thing, this is a huge piece in making sure the patient gets better and certainly, if I feel confident in the way I'm treating you you're going to feel more like you're in the right place to be treated.” (P21)

Participants were disappointed that they did not gain more confidence in their ability to understand statistical methods. Low self-efficacy around this topic was troubling to participants and many expressed a desire to pursue further education.

“I would have to say that my ability to look at statistics is not as strong as I would like it to be even still.” (P20)

#### Knowledge and skills

The PEAK program was not associated with any change in knowledge and skills as measured by the mFT (p = 0.10; Table 2). After the program, the cohort had comparatively high scores for knowledge and skills associated with searching (62-84% of possible points) and low scores for items requiring statistical knowledge and skill (5-29% of possible points).

With regard to knowledge, the most notable participant comments related to insufficient statistical knowledge.

“So I think it’s very important, but I'll have to admit, I don't know how to analyze it properly when you get into the detailed aspects of statistics.” (p15)

With regard to skills, participants described the PEAK program as providing a valuable opportunity to improve through practice. For example, participants described learning to ask better questions as a basis for their searching.

“Oh, I'm so much better. I've revised all the way I've done the clinical questions. And so that's been kind of a learning opportunity for me.” (F5)

Participants also described identifying better search terms, using newly learned features in PubMed, and cross-referencing results from more than one search engine.

“…learning how to narrow my questions…initially I thought I was missing out on a lot, but now it’s very focused.” (P19)

Other new skills included searching specifically for clinical practice guidelines and systematic reviews.

“My approach has changed in that often I'm trying to find clinical guidelines in different areas of treatment to get a broad array of, what are the approaches that current experts in the field are taking?” (F15)

They identified a pattern where, as skills improve, confidence increases and skills are further enhanced through more practice.

“Being able to go onto a database like Pub Med and have a more effective, efficient search was very helpful…the more efficient you are at something, the less time you spend doing it, the more apt you are to do it again.” (P14)

Participants also described developing their skills of critical appraisal as they expressed new insights into the strengths and weaknesses associated with different study designs.

“Being a more savvy consumer of research, looking at methods and results, is this a reliable and valid study, does it match my patient population, is their sample size large enough, how did they select, include or exclude their test subjects, to be able to look at a strength of a study based on that, and then to make my independent assessment of the results without having the author's coloring of the data, I think has been very nice.” (P20)

#### Self-reported behaviors

The PEAK program was associated with a 20.4% increase in mean score for 13 items of the EBP Implementation Scale (p = 0.001; Table 2). Post-hoc analyses showed that 7 items had statistically significant increases from baseline (item number and topic): 2 and 7 - reading and critically appraising research studies; 3 - generating PICO questions; 6 - presenting evidence to >2 colleagues; 8 - sharing an EBP guideline with a colleague; 10 - sharing evidence with a multi-disciplinary team; 12 - accessing Cochrane Systematic Reviews; and 13 - accessing National Guidelines Clearinghouse).

Participants described integrating the research evidence more directly in patient care and in discussions with patients.

“I find more that I’m incorporating it in my education of the patients. I’m always talking to them. This is why we’re doing this and so on. And I feel like they really appreciate that. Or I’ll use it to ask them, ‘these are the recommendations, what do you really prefer?’” (F5)

Participants also described discussing ways in which they could integrate research evidence into their clinical practice more with their peers.

“I see people sitting around the table discussing articles, sharing articles, and people that weren't involved in that previously are now involved.” (P2)

Lastly, participants described active reflection about the process of using evidence in their clinical decision-making.

“Let me figure out why I haven't done that with my last patient and see if there's a reason. So now I can check myself and see, am I doing those things, am I not doing those things, why or why not and what is the evidence that supports it?” (F7)

#### Patient-oriented outcomes

Although patient-oriented outcomes were not assessed quantitatively, participants described feeling that the process of developing the Best Practices List would result in more accurate and evidence-based clinical decisions for patients with lumbar spine conditions. Ultimately, they believed that patients would experience better outcomes.

“You’re putting the patients’ needs first, you’re using your own past experience just to manage the patient, and then you're using the top evidence. So I think that would be the key and I think then your average patient would want to participate or have a clinician treating and using those standards” (P14)

Participants also believed that inclusion of a common set of outcome measures in the Best Practices List would support evidence based clinical decision-making, improve patient education, and enhance communication with other healthcare professionals.

“We’ve started kind of monthly in-services and having discussion. What’s the latest within the literature, what are the recommended outcome measures we should be using for these various patient populations?” (P3)

### Best Practices List

Participants selected the topic of ‘Lumbar Spine Conditions’ with five sub-topics (outcome measures, stenosis, spine tumors, non-specific low back pain, and post-surgical) for which they reviewed the literature and identified locally relevant, actionable, evidence-based behaviors that should be implemented in their practice. At the conclusion of the program, participants had created a Best Practices List consisting of 38 evidence-based behaviors (see Additional file 1) drawn from clinical practice guidelines, systematic reviews, randomized controlled trials, cohort studies, case series, and narrative reviews. Fourteen participants (77%) rated themselves as being ‘Involved’ or ‘Very Involved’ in development of the Best Practices List.

### Explanatory themes from mixed methods analysis

Thus far, the qualitative data supported and provided a deeper explanation of the changes observed in quantitative scores. Four explanatory themes emerged from the mixed-methods analysis to characterize the feasibility of the PEAK program.

1. **The collaborative nature of PEAK was engaging and motivating.**

Participants were engaged from the beginning of the PEAK program in working in small groups to search for and appraise literature around topics of interest. Most small groups interacted regularly and new patterns of collaboration developed across the different clinical sites, all with a focus on understanding and integrating research evidence into practice. Collaboration was facilitated by the range of online collaboration tools, regular meetings, and the ultimate goal of agreeing on the Best Practices List. Technologic resources for collaboration were generally successful, however, some participants found it difficult to learn to use these tools. Participants were particularly motivated to collaborate by anticipation of patient benefit.

2. **PEAK participants experienced improved self-efficacy which created a positive cycle where success reinforced engagement with the research evidence.**

Participants demonstrated, and almost all described, improved confidence and capacity, most notably for searching for research evidence. This new found confidence fueled a positive trend that led to increased confidence and engagement in utilizing research evidence in their clinical practice. In addition, this increased confidence appeared to reinforce the benefit of spending what was often personal time engaged in the PEAK program. It was reinforcing for participants to search for and find relevant research evidence that they could discuss with peers and patients. Ultimately, this process led to improved confidence and perceived benefit to patients.

3. **Participants’ need to understand how to interpret statistics was not fully met by the PEAK program.**

Participants were motivated to appraise and interpret research evidence in order to integrate research results into their own practice. They were quick to recognize the limitations of published research, and to make comparisons with their own patient populations. However, many identified limitations in their statistical knowledge and skills and expressed disappointment that they did not gain more confidence and skill in this area through the PEAK program. Participant self-efficacy and knowledge and skills around this topic were substantially lower than any other element of EBP. Likewise, this was the predominant negative theme in interviews and focus groups.

4. **Participants believed that the process of using relevant research evidence to develop the Best Practices List would lead to better patient outcomes.**

Participants felt that the process of identifying and appraising research evidence to develop the Best Practices List would lead to better clinical outcomes for patients. They emphasized the practical benefits of developing consistent and routine patterns of care that were informed by research evidence. Furthermore, participants described being able to provide more effective care with higher confidence – both elements they expected would have positive effects on patient outcomes. Finally, they anticipated improved continuity of care as they all agreed to use the locally generated Best Practices List in their clinical practice.

## Discussion

The PEAK program is a feasible educational program for promoting physical therapist use of research evidence to inform clinical practice across three clinical sites in a university-based healthcare system. All participants completed the 6-month educational program and most reported high levels of involvement. The group developed, and agreed to implement, a Best Practices List consisting of 38 evidence-based behaviors around caring for individuals with lumbar spine conditions. Participants’ reaction to the PEAK program was consistently positive and quantitative measures demonstrated that the program was associated with improvements in EBP self-efficacy and self-reported behaviors. Four themes from our mixed-methods analysis provide insight into the program and implications for its future use around the topics of: benefits of the collaborative nature of the program, improved self-efficacy for integrating research evidence, need for more detailed understanding of statistics, and belief that patient care was improved by informing clinical practice with research evidence.

Most physical therapy-specific KT studies have focused on changing clinical decision-making around a single clinical practice guideline [26-29] or pre-packaged evidence summary [30]. Two have focused on development of more generalizable EBP and KT skills [4,31]; both reported limited change in therapist outcomes. The PEAK program addresses the need for physical therapists to use a wide variety of resources (as opposed to a single clinical practice guideline) to support clinical decision-making. In addition, it addresses not only individual-level barriers to EBP but also takes into account the need to address organizational resources and cultural issues to support KT across the continuum of care in a dispersed healthcare system.

The PEAK program’s foundation in social cognitive theory [8] offers an explanation for the individual change observed among participants. By using small groups to generate a sense of community, participants felt engaged and motivated to use the knowledge and skills they had gained to search for and critically appraise the research evidence. They accepted verbal knowledge from a credible source during the 2-day workshop, observed each other searching for and critically appraising key journal articles, and experienced personal success through guided learning. Each of these elements is likely to have motivated participants to repeat their behaviors [32], ultimately leading to successful completion of the Best Practices List and the self-reported increase in use of research evidence in practice.

The use of adult learning theory concepts [9] resulted in a program that was driven by participants, for their own practical benefit. Participants selected the topic for the Best Practices List and self-selected into small groups that worked independently towards meeting an immediate clinical need. Similarly participants reported that the creation of the Best Practices List was the most important part of the program and that they were motivated by a commitment to provide high quality patient care. It is likely that this helped to generate a sense of ownership in the process. Further, use of the PARiHS [10] and Knowledge to Action [11] Cycle frameworks drove elements of the program that were deemed important by participants, including: leadership support, provision of resources, and emphasis on adapting research evidence to support local needs.

Despite the feasibility of the program, we learned several lessons that we expect will improve future versions. Most importantly, qualitative and quantitative data strongly suggest that participants needed additional knowledge and skills to understand and interpret statistics. Although the 2-day workshop and monthly meetings included some education around statistics and interpretation of results, it was clearly insufficient. This challenge must be met with sensitivity to the fact that it may not be feasible to expect clinicians to become experts in statistics. We also learned that some participants needed more assistance with technologic resources and that while monthly meetings for supplemental education and discussion were valuable, poor performance of our video conferencing system was frustrating for all.

This study is the first, to our knowledge, to use the CREATE model [19] as a foundation for assessing EBP learning. The CREATE model provided a cohesive method for evaluating the complexity of component interventions within our educational program. By comparing quantitative and qualitative results across the CREATE framework we gained a deeper understanding of which components were valued by participants, and how these contributed to improved self-report scores. Based on the early work by Kirkpatrick [33], the CREATE assessment categories are expected to build on each other – from the most direct impact (reaction to the educational program) to the most complex (improving patient outcomes) [19]. Yet, although participants experienced quantitative change in self-efficacy and self-reported behavior, we did not observe a quantitative change in the intervening categories of knowledge and skills. Qualitative data suggest that while knowledge may not have changed, participants’ felt that their skills in searching for, appraising, and integrating research evidence into practice had improved. This suggests that the mFT may be an insufficient tool for identifying changes in EBP skills distinct from EBP knowledge. Furthermore, while we did not assess change in patient outcomes, therapists felt strongly that their patients had benefited. This supports future work to assess patient reported outcomes and clinical improvement in association with therapist participation in PEAK.

### Limitations

This study has four important limitations. First, from the perspective of quantitative results, the number of participants was small. Although the population was relatively diverse (age, years of experience, degrees, clinical setting), a larger sample size with a subset used for the qualitative analysis would have been a stronger design. Second, the participant population lacked diversity in that they all worked at USC. There is a selection bias among individuals who pursue, and get the opportunity to, work at a university teaching hospital or clinic. While previous studies have established that physical therapists routinely report strongly positive attitudes about EBP [2,15,34], our volunteer participants may represent the far end of the spectrum for positive attitudes. Additionally, and perhaps more importantly, all participants had access to a high quality medical library and medical librarian. Replication of the PEAK program without full-text access to most rehabilitation journals will pose an additional challenge. Third, this analysis does not assess long term outcomes. Further study is needed to determine whether improvements associated with participation were sustained and whether the Best Practices List was effectively implemented in patient care. Finally, two of the standardized quantitative assessment tools (EBP Belief Scale and EBP Implementation Scale) were modified to ensure that a single domain (EBP attitudes and behavior for using research evidence, respectively) was being assessed. While the items used from each tool had strong face validity, neither was validated in their abbreviated format. We felt that these modifications were reasonable for a feasibility study given that better, single domain, tools were not available. However, this is an important area for development to support further investigations of the PEAK program and implementation research in general.

Finally, it is important to note that the PEAK program was designed to influence one component of clinical decision-making—the integration of research evidence. Clinical decision-making is influenced by a complex host of issues (e.g. culture, emotion, moral, political, etc.) and often involves tensions between scientific reason and social reality [35]. While the PEAK program addressed the integration of research evidence with patient perspective, it did not explicitly address the broader context of collaborative and patient-centered shared decision-making.

## Conclusion

The multifaceted, learner-centered PEAK program is a feasible educational program for promoting physical therapists’ use of research evidence in clinical decision-making. Four themes informing feasibility of the program relate to 1) the collaborative nature of the program; 2) improved self-efficacy for using research evidence to inform practice; 3) the need for greater learning around statistics; and 4) participant expectation that the list of evidence-based practices developed and agreed to by the group would lead to better patient outcomes. A key ingredient seems to be guided small group work leading to a final product (Best Practices List) that guides local practice. Further investigation is recommended to assess long-term behavior change and to compare outcomes to alternative educational models.

## Appendix A

### Core interview template

Introduction, Reaction to the EBP Experience:

What small group were you in?

How was the Fellowship?

What aspects were most helpful to you?

How has your work environment impacted on your ability to engage in the Fellowship?

How have you used the librarian and library resources?

Knowledge about EBP principles:

Has the Fellowship influenced the way you think about and interpret research?

Has there been a change in how you use clinical guidelines in your practice?

Skills for performing EBP:

Has the Fellowship taught you new skills?

[if YES] How did the Fellowship help you learn these skills?

How do you keep up to date with the research evidence?

Behavior as part of patient care:

Has this Fellowship impacted on the way you work with your patients?

Has the Fellowship influenced the way you work with your colleagues?

Self-Efficacy for conducting EBP:

In what way has this Fellowship changed your ability to use research evidence (ask across 5 steps)?

What will you do differently, as a result of participating in this Fellowship?

Benefits to patients associated with EBP training:

Have there been any benefits for your patients, from your involvement in the Fellowship?

Attitudes about EBP:

Does EBP impact on the way you work with your patients?

What is the most important aspect of EBP in your clinical practice?

Future Thinking:

If you could change one thing in your current work to improve EBP, what would it be?

If this Fellowship were to be expanded to over 100 people, what would you do differently?

## Competing interests

The authors declare that they have no competing interests.

## Authors’ contributions

JKT designed the PEAK program, developed the overarching study design, collected and analyzed quantitative data, and had primary responsibility for drafting the manuscript; SM developed the qualitative study design, conducted qualitative interviews, directed analysis of qualitative data, and made major contributions to drafting the manuscript; JCS, MZ, JMD, and RH made substantial contributions to the study design and acquisition of data and critically reviewed the manuscript for important intellectual content. All authors gave final approval of the manuscript.

## Pre-publication history

The pre-publication history for this paper can be accessed here:

http://www.biomedcentral.com/1472-6920/14/126/prepub

## Supplementary Material

Additional file 1 Best Practices List generated by PEAK participants: “University of Southern California Best Practices List: Physical Therapy for Lumbar Spine Conditions”. Click here for file
